# Effects of Telemedicine and mHealth on Systolic Blood Pressure Management in Stroke Patients: Systematic Review and Meta-Analysis of Randomized Controlled Trials

**DOI:** 10.2196/24116

**Published:** 2021-06-11

**Authors:** Meina Lv, Tingting Wu, Shaojun Jiang, Wenjun Chen, Jinhua Zhang

**Affiliations:** 1 Department of Pharmacy Fujian Medical University Union Hospital Fujian Medical University Fuzhou China

**Keywords:** stroke, systolic blood pressure, mHealth, telemedicine, meta-analysis, self-management

## Abstract

**Background:**

Stroke is a common, harmful disease with high recurrence and mortality rates. Uncontrolled blood pressure is an important and changeable risk factor for stroke recurrence. Telemedicine and mobile health (mHealth) interventions may have the potential to facilitate the control of blood pressure among stroke survivors, but their effect has not been established.

**Objective:**

This systematic review and meta-analysis of randomized controlled trials (RCTs) was conducted to estimate the effects of telemedicine and mHealth interventions on the control of systolic blood pressure among stroke survivors.

**Methods:**

The research literature published up to June 28, 2020, and consisting of RCTs related to telemedicine and mHealth interventions was searched in PubMed, EMBASE, Web of Science, and the Cochrane Library. The Cochrane risk of bias tool (RoB 2.0) was used to evaluate the quality of the studies. The Cochran *Q* test and *I*^2^ statistic were used to assess heterogeneity. Data were meta-analyzed using a random-effects model. Mean difference (MD) with 95% CI and 95% prediction interval (PI) were calculated.

**Results:**

In total, 9 RCTs with a total sample size of 1583 stroke survivors met the inclusion criteria. Compared with the usual care, telemedicine and mHealth had a significantly greater impact on the control of systolic blood pressure (MD –5.49; 95% CI –7.87 to –3.10; *P*<.001; 95% PI –10.46 to –0.51). A subgroup analysis showed that the intervention mode of telephone plus SMS text messaging (MD –9.09; 95% CI –12.71 to –5.46; *P*<.001) or only telephone (MD –4.34; 95% CI –6.55 to –2.13; *P*<.001; 95% PI –7.24 to –1.45) had a greater impact on the control of systolic blood pressure than usual care. Among the stroke survivors with an intervention interval ≤1 week (MD –6.51; 95% CI –9.36 to –3.66; *P*<.001; 95% PI –12.91 to –0.10) or a baseline systolic blood pressure ≥140 mm Hg (MD –6.15; 95% CI –9.44 to –2.86; *P*<.001; 95% PI –13.55 to 1.26), the control of systolic blood pressure using telemedicine and mHealth was better than that of usual care.

**Conclusions:**

In general, telemedicine and mHealth reduced the systolic blood pressure of stroke survivors by an average of 5.49 mm Hg compared with usual care. Telemedicine and mHealth are a relatively new intervention mode with potential applications for the control of systolic blood pressure among stroke survivors, especially those with hypertensive stroke.

## Introduction

Stroke is a common, harmful disease and a main cause of death and disability worldwide. It has the characteristics of high morbidity, disability, recurrence, and mortality [1-3]. Stroke survivors have a high risk of recurrence, with recurrent stroke entailing more severe symptoms and worse results than the first occurrence [4,5]. Stroke not only affects patients’ quality of life but also imposes an economic burden on the family, medical system, and society [6,7]. However, about 85% of stroke cases are preventable, and effective secondary prevention can reduce the recurrence rate of stroke [8-10].

Noncommunicable diseases are the main causes for the increase in the incidence of stroke. Approximately 90.5% of global stroke diseases can be attributed to modifiable risk factors, among which hypertension is the most common for first and recurrent strokes but is modifiable [11-13]. Uncontrolled blood pressure is an important changeable risk factor for stroke recurrence. Implementing secondary preventive measures can reduce the recurrence of stroke by 80% [14]. A recent systematic review and meta-regression analysis emphasized that strict and active blood pressure control may be the most critical treatment strategy for the secondary prevention of stroke, highlighting that the reduction in systolic blood pressure is linearly related to reduction in the risk of recurrent cerebrovascular events [15]. However, many stroke survivors have the risk factor of high blood pressure [14]. More than one-third of patients continue to have poor blood pressure control following a stroke or transient ischemic attack, but most people are unaware of these risks [14,16].

Therefore, some researchers have tried to reduce the risk of recurrent cerebrovascular events through interventions to improve blood pressure after a stroke or transient ischemic attack. Telemedicine and mobile health (mHealth) interventions have a potential role in this endeavor. An increasing number of studies have been conducted on the use of telemedicine and mHealth interventions to manage systolic blood pressure in stroke survivors [17-25], but it is not clear whether their effect is better than that of usual care. To objectively evaluate the efficacy of these interventions and provide a reference for clinical application, this study adopted the Cochrane evaluation method to conduct a systematic review and meta-analysis of existing international randomized controlled trials (RCTs) related to telemedicine and mHealth for control of systolic blood pressure in stroke survivors.

## Methods

### Data Sources and Search Strategy

This study follows the PRISMA (Preferred Reporting Items for Systematic Reviews and Meta-Analyses) guidelines [26]. We conducted a comprehensive literature search in online databases, including PubMed, EMBASE, Web of Science, and the Cochrane Library. In order to conduct a comprehensive search, we also searched Chinese literature, gray literature, and the reference lists of the studies yielded by the original search. We searched relevant studies published until June 28, 2020. The search keywords were as follows: “stroke” OR “brain infarction” OR “transient ischemic attack” OR “cerebral hemorrhage” OR “subarachnoid hemorrhage,” “mobile applications” OR “telemedicine” OR “text messaging” OR “cell phone” OR “smartphone” OR “social media” OR “internet,” and “blood pressure” OR “hypertension.” A detailed search strategy for each database is presented in Multimedia Appendix 1. The literature search and screening were carried out independently by 2 researchers (ML and TW).

### Inclusion Criteria

We included all studies that met the following requirements: the study’s design was an RCT, participants were diagnosed with a stroke (hemorrhagic stroke or ischemic stroke) or transient ischemic attack, interventions were provided for patients using telemedicine (with telemedicine defined as the provision of health services at a distance using a range of technologies, such as telephone, telemonitoring, etc [27,28]) and mHealth (with mHealth defined as the delivery of health service through mobile and wireless applications, including mobile phones, SMS text messaging, wearable devices, etc [29]), the control group received usual care, and the main outcome indicator was systolic blood pressure.

### Exclusion Criteria

Studies were excluded from the meta-analysis if any of the telemedicine and mHealth intervention or usual care management was independently discussed, or if the original research data were incomplete or unusable and useful data could not be obtained by contacting the original author.

### Data Extraction

The data were retrieved from the selected studies. The extracted data included study information (author, publication year, country), study characteristics (study population, sample size), participants’ characteristics (age, gender, baseline systolic blood pressure), intervention information (intervention mode, intervention interval), and main outcome indicators (systolic blood pressure). The required data were extracted independently by 2 researchers (ML and TW) and cross-referenced to avoid potential extraction errors. All disagreements were discussed with a third researcher to reach a consensus.

### Quality Assessment

Two independent researchers used the Cochrane risk of bias tool (RoB 2.0) [30] to evaluate the quality of the selected literature. The items addressed were as follows: bias arising from the randomization process, deviations from intended interventions, bias due to missing outcome data, bias in measurement of the outcome, bias in the selection of the reported result, and overall risk of bias. An additional researcher was asked to conduct an evaluation to help resolve disputes that arose during the evaluation process.

### Statistical Analysis

Stata version 14.0 (StataCorp) was used for the meta-analysis. The Cochran *Q* test and *I*^2^ statistic were used to assess heterogeneity [31]. In the heterogeneity assessment, *I*^2^ is considered to be nil if it is below 25%, low if it is 25%-50%, moderate if it is 51%-75%, and high if it is above 75% [32]. Due to expected heterogeneity (study characteristics and the manner in which studies were conducted) between studies, a random-effects model was used to estimate the mean difference (MD) with 95% CI being considered the statistic of interest [33]. In addition, the 95% prediction interval (PI) was calculated for the overall weighted mean estimate [34]. To explore the factors influencing mHealth interventions, we conducted subgroup analyses of the intervention mode, intervention interval, and baseline systolic blood pressure. Interrater agreement was calculated by using the κ statistic according to the following scheme: κ value <0, worse than that expected by chance; 0.21-0.40, poor; 0.41-0.60, moderate; 0.61-0.80, good; and 0.81-1.00, very good level of agreement [35]. Publication bias was evaluated by inspection of funnel plots and Egger tests [36]. In this study, a *P* value <.05 was considered statistically significant.

## Results

### Study Selection

**Figure 1:**
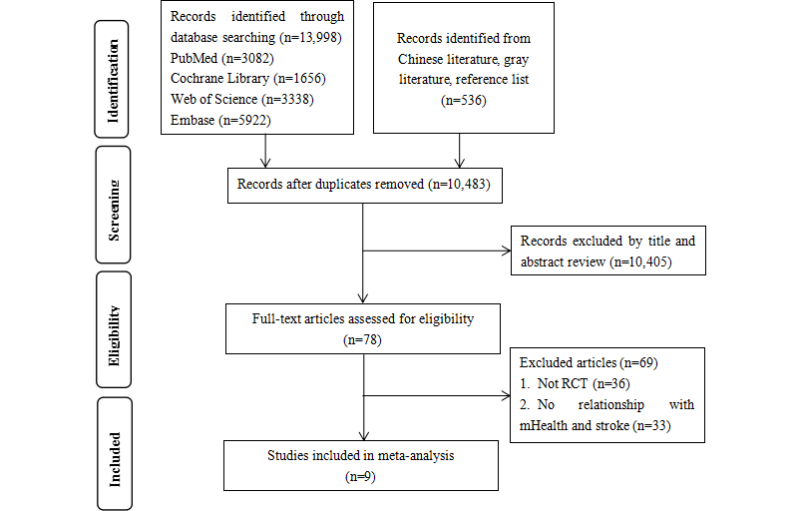
Flow diagram of the selection of studies. mHealth: mobile health; RCT: randomized controlled trial.

A total of 13,998 studies were retrieved using the search strategy. After screening, 9 studies were included in the meta-analysis, comprising a total of 1583 patients: 798 in the mHealth intervention group and 785 in the usual care group. The literature screening process and results are shown in Figure 1.

### Study Characteristics and Quality Assessment

The basic characteristics of the included studies are presented in Multimedia Appendix 2. All of the included studies were RCTs and published in 2010 or later, which is in line with the rapid development and spread of mHealth technology in recent years. These studies were conducted in different countries and regions, of which 3 were from the United Kingdom [17-19], 2 from the United States [20,21], 2 from China [23,24], 1 from Ghana [22], and 1 from Sweden [25]. The participants were stroke survivors. The mean or median age of the patients ranged from 54.3 years to 73.5 years. The proportion of women ranged from 23.1% to 60.0%. We used the Cochrane risk of bias tool (RoB 2.0) to evaluate the risk of bias in the 9 included studies. The results showed that risk of bias was deemed to be either “low” or “with some concerns” (Table 1). A κ value of 0.768 (95% CI 0.673-0.841; *P*<.001) in this study indicated that there was a good agreement between encoders.

**Table 1:** Cochrane risk-of-bias tool for randomized controlled trials (RoB 2.0).

Study	Bias arising from the randomization process	Deviations from intended interventions	Bias due to missing outcome data	Bias in measurement of the outcome	Bias in selection of the reported result	Overall risk of bias

Adie et al (2010) [17]	Low	Low	Low	Low	Low	Low^a^
Hanley et al (2015) [18]	Some concerns	Some concerns	Low	Low	Low	Some concerns^b^
Kerry et al (2013) [19]	Low	Low	Low	Low	Low	Low
Lakshminarayan et al (2018) [20]	Low	Some concerns	Low	Low	Low	Some concerns
Mackenzie et al (2013) [21]	Low	Some concerns	Low	Low	Some concerns	Some concerns
Sarfo et al (2018) [22]	Low	Low	Low	Low	Low	Low
Wan et al (2018) [23]	Low	Low	Low	Low	Low	Low
Wang et al (2020) [24]	Low	Low	Low	Low	Low	Low
Ögren et al (2018) [25]	Low	Some concerns	Some concerns	Low	Low	Some concerns

^a^Low: when present in this column, this indicates the study is judged to be at low risk of bias for all domains for this result.

^b^Some concerns: when present in this column, the study is judged to raise some concerns in at least one domain for this result, but not due to a high risk of bias for any domain.

### Comparison of Changes in Systolic Blood Pressure

**Figure 2:**
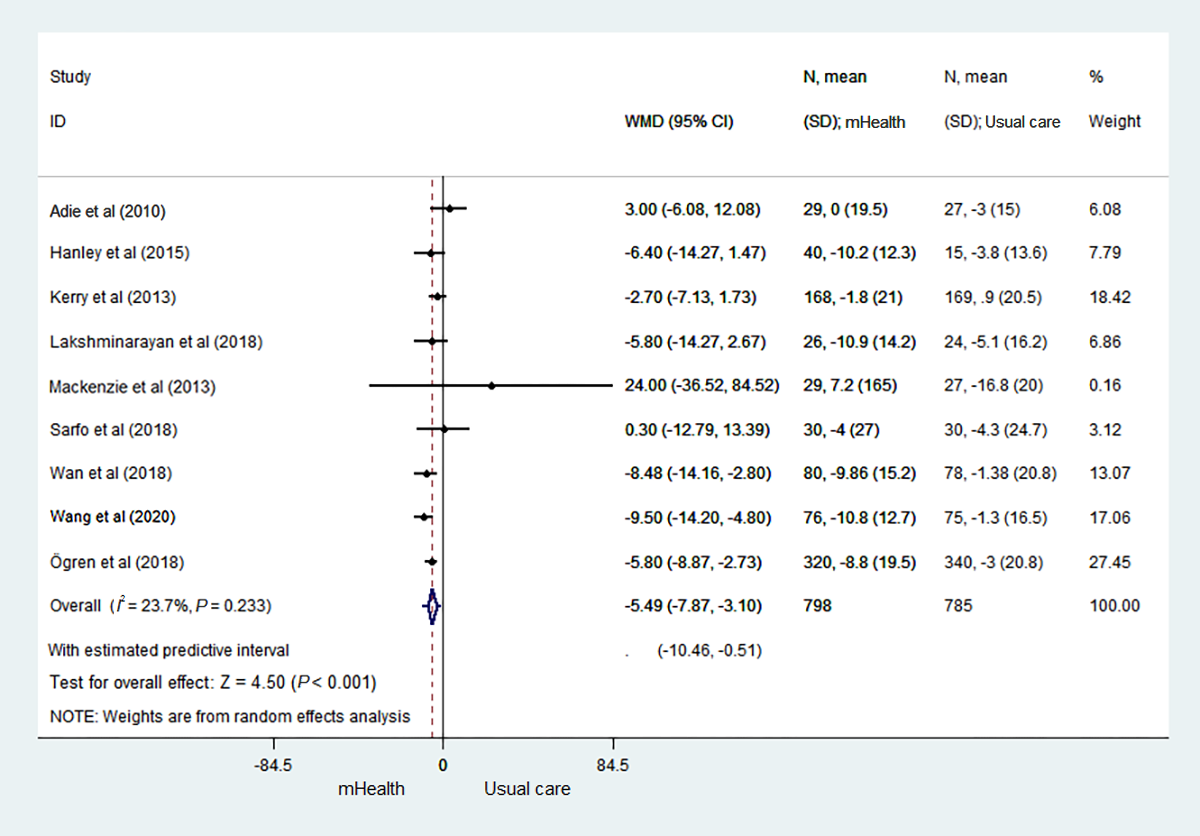
Forest plot of the systolic blood pressure of the telemedicine and mHealth group and usual care group. mHealth: mobile health; WMD: weighted mean difference.

Figure 2 [17-25] illustrates the changes in systolic blood pressure between the 2 groups in the 9 studies. There was statistical heterogeneity between studies (*I*^2^=23.7%). The results showed that the control of systolic blood pressure of the stroke survivors in the telemedicine and mHealth group was better than that of the stroke survivors in the usual care group, and the difference was statistically significant (MD –5.49; 95% CI –7.87 to –3.10; *P*<.001; 95% PI –10.46 to –0.51).

### Subgroup Analyses

**Figure 3:**
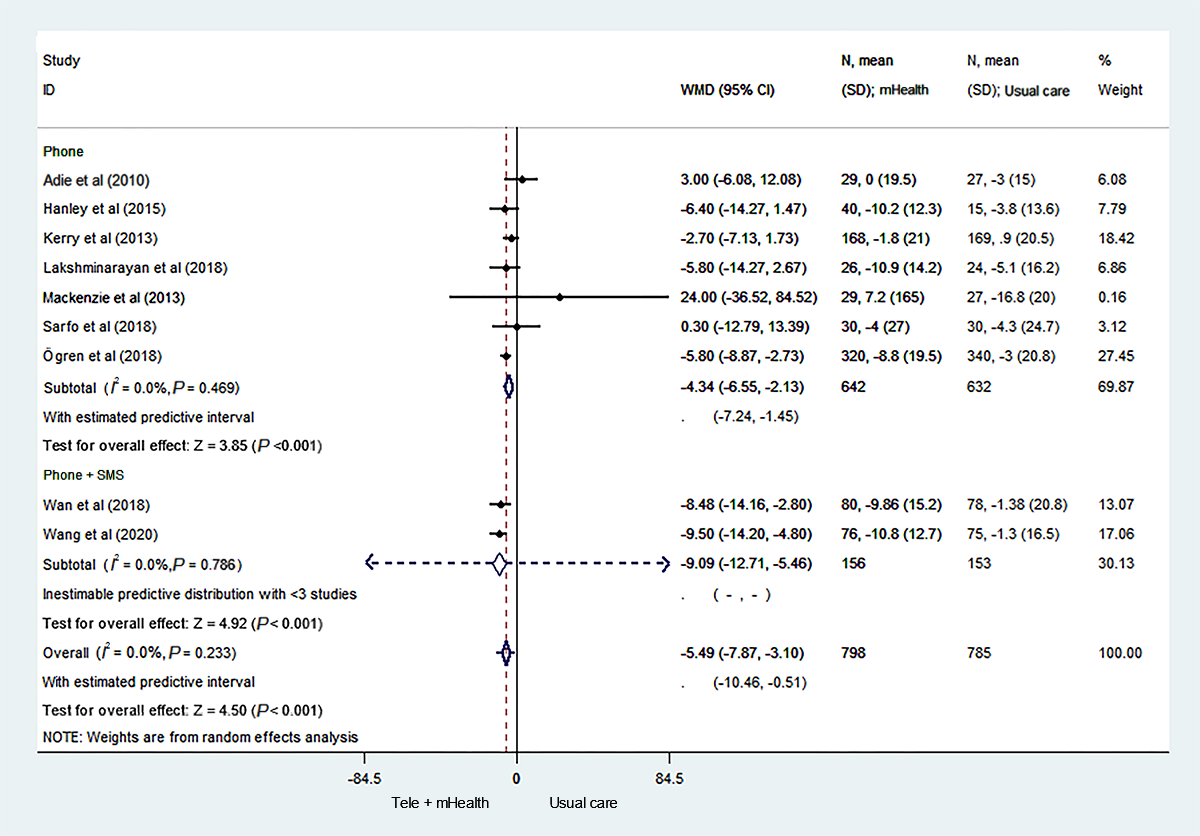
Forest plot of the subgroup analysis of the mode of intervention. mHealth: mobile health; WMD: weighted mean difference.

We conducted subgroup analyses of the intervention mode. When the intervention mode consisted of telephone plus SMS text messaging or only telephone, the telemedicine and mHealth group showed a larger effect on the control of systolic blood pressure than did the usual care group, with an MD of –9.09 (95% CI –12.71 to –5.46; *P*<.001) and –4.34 (95% CI –6.55 to –2.13; *P*<.001; 95% PI –7.24 to –1.45), respectively (Figure 3 [17-25]).

**Figure 4:**
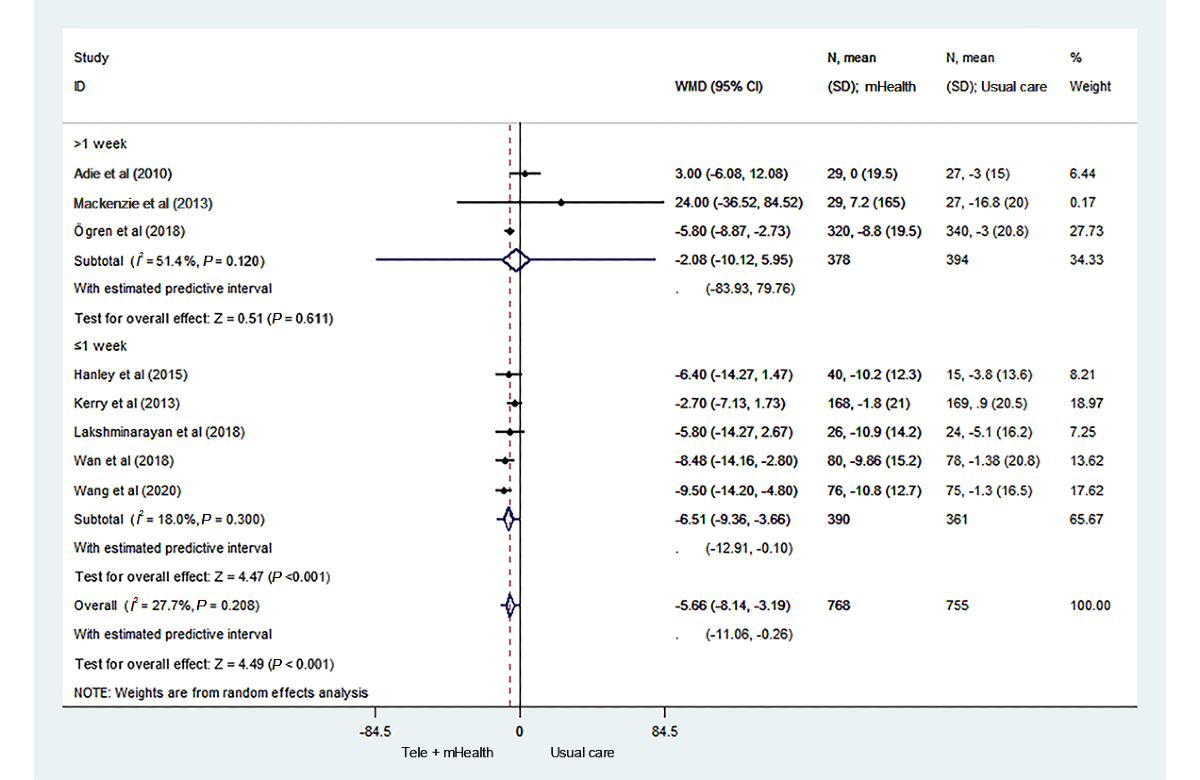
Forest plot of the subgroup analysis of the intervention interval. mHealth: mobile health; WMD: weighted mean difference.

**Figure 5:**
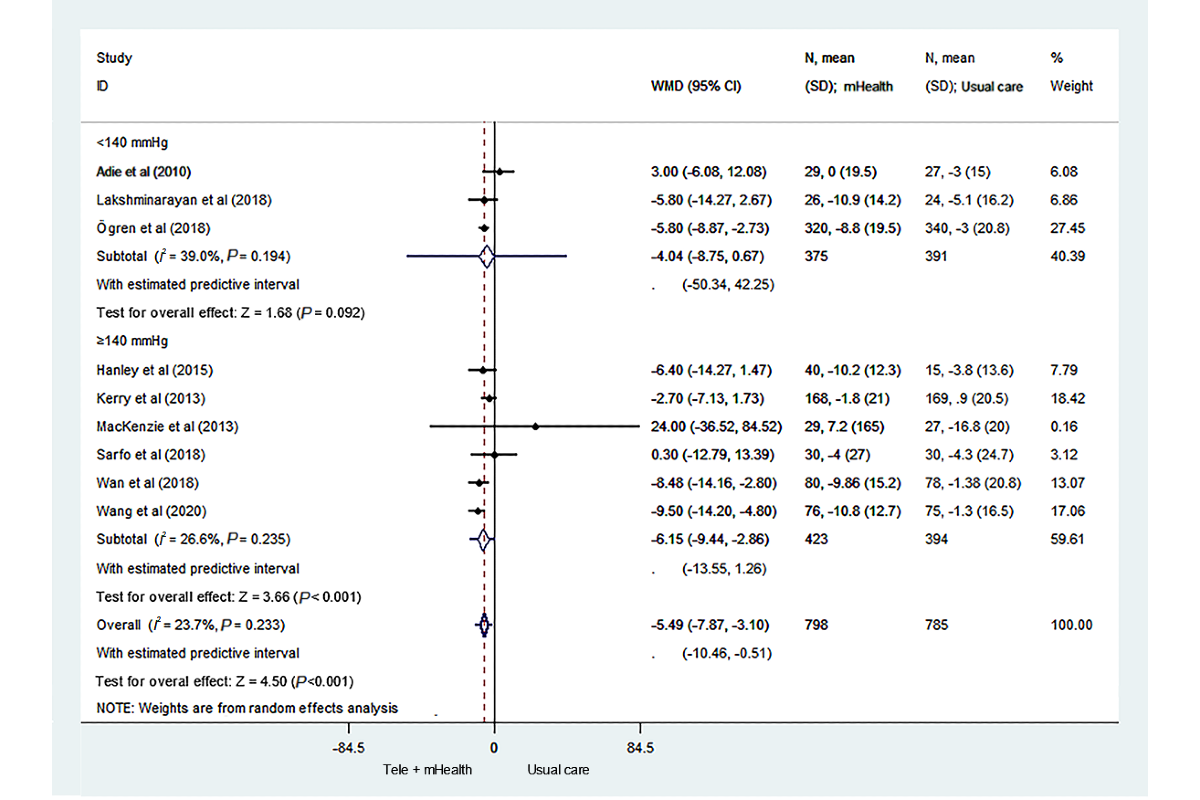
Forest plot of the subgroup analysis of the baseline systolic blood pressures. mHealth: mobile health; WMD: weighted mean difference.

We also performed subgroup analyses of the intervention interval and baseline systolic blood pressure (Figures 4 and 5 [17-25]). Compared to the usual care group, the telemedicine and mHealth group had better control of systolic blood pressure, with an intervention interval ≤1 week, and the difference was statistically significant (MD –6.51; 95% CI –9.36 to –3.66; *P*<.001; 95% PI –12.91 to –0.10). When the intervention interval was greater than 1 week, no significant difference was found in the control of systolic blood pressure between the 2 groups (MD –2.08; 95% CI –10.12 to 5.95; *P*=.61; 95% PI –83.93 to 79.76). In addition, among the stroke survivors with a baseline systolic blood pressure <140 mm Hg, no significant difference in the control of systolic blood pressure was found between the mHealth intervention group and the usual care group (MD –4.04; 95% CI –8.75 to 0.67; *P*=.09; 95% PI –50.34 to 42.25). In contrast, among the stroke survivors with a baseline systolic blood pressure ≥140 mm Hg, the control of systolic blood pressure of the telemedicine and mHealth group was significantly better than that of the usual care group, and the difference was statistically significant (MD –6.15; 95% CI –9.44 to –2.86; *P*<.001; 95% PI –13.55 to 1.26).

### Publication Bias and Sensitivity Analysis

**Figure 6:**
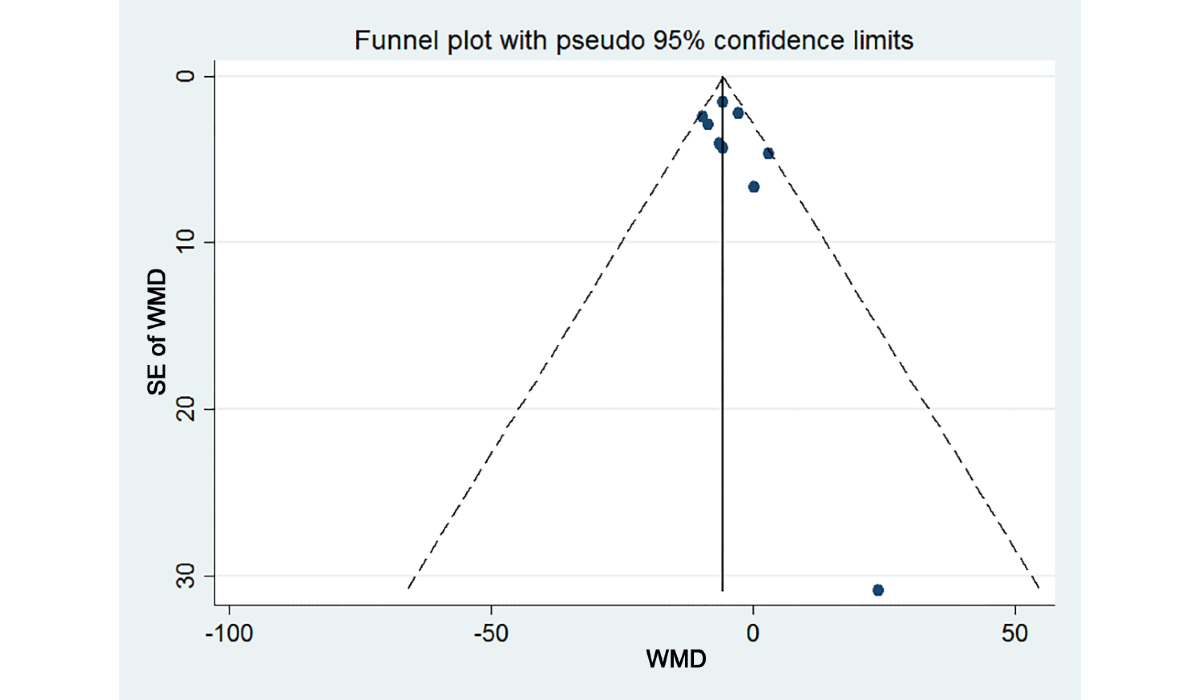
Funnel plot of the systolic blood pressures. WMD: weighted mean difference.

Funnel plot inspection and the Egger test showed no publication bias (*P*=.16; Figure 6). Furthermore, a sensitivity analysis of the outcome indicators of systolic blood pressure was conducted using the method of excluding relevant studies one by one. The results did not change significantly, indicating that the findings of this analysis were stable.

## Discussion

Stroke is characterized by high recurrence and mortality rates [1-3]. Hypertension is an important risk factor for stroke recurrence [13], so it is essential for stroke survivors to control their blood pressure. The main purpose of this meta-analysis was to evaluate the effect of telemedicine and mHealth interventions on the control of systolic blood pressure in stroke survivors. We conducted a systematic review and meta-analysis of 9 RCTs. Compared with usual care, the telemedicine and mHealth intervention reduced the systolic blood pressure by an average of 5.49 mm Hg. It is worth mentioning that this change in systolic blood pressure was equivalent to the decrease in the systolic blood pressure reported in a meta-analysis of interventions to improve the lifestyles of patients (eg, reasonable diet, aerobic exercise, restriction of alcohol and sodium intake) [37,38]. Studies have shown that a 3-mm Hg reduction in systolic blood pressure can reduce stroke mortality by 8% [39]. Therefore, telemedicine and mHealth interventions for stroke survivors may be a measure worth considering.

As far as we know, this is the first systematic, quantitative analysis and summary of all available evidence of telemedicine and mHealth interventions for the management of systolic blood pressure in the population of stroke survivors. More importantly, we found that the stroke survivors in the telemedicine and mHealth group had better control of their systolic blood pressure than did the usual care group after receiving interventions that actively sent electronic messages (telephone calls, SMS text messages). This result may be expected because poor self-management and poor compliance are major problems affecting patients’ blood pressure control [40]. Stroke survivors with low compliance may benefit more from active interventions, such as telephone calls or SMS text messages. Therefore, active telemedicine and mHealth interventions may yield clinical benefits for stroke survivors by helping them achieve blood pressure control.

This meta-analysis included RCTs from different countries (the United States, the United Kingdom, Sweden, Ghana, and China), indicating that mHealth interventions may be applicable to people in different countries and different medical systems. Furthermore, the average baseline systolic blood pressure was 128.0-154.0 mm Hg, which indicates that the included studies targeted stroke survivors extensively for telemedicine and mHealth interventions. We found that among the stroke survivors with a baseline systolic blood pressure <140 mm Hg, there was no significant difference between the telemedicine and mHealth group and the usual care group. However, for stroke survivors with a baseline systolic blood pressure ≥140 mm Hg, the telemedicine and mHealth group had significantly better control of systolic blood pressure than did the usual care group. This is a major finding in stroke survivors with a baseline systolic blood pressure ≥140 mm Hg, which indicates that telemedicine and mHealth interventions may have greater benefits for stroke survivors with hypertension. If the proper intervention is conducted for an extended period, this may have a significant clinical impact.

Telemedicine and mHealth interventions are becoming an increasingly common way to support patients with chronic diseases in adhering to their medications and conducting self-management [41]. Telemedicine and mHealth interventions can provide reminder strategies and help patients achieve self-monitoring of blood pressure to improve their medical and behavioral management. Nursing staff can make personalized recommendations for blood pressure management based on patients’ feedback. We found that when the intervention interval was ≤1 week, the influence on the control of systolic blood pressure of the telemedicine and mHealth group was significantly greater than that of the usual care group. However, there was no significant difference between the 2 groups when the intervention interval was more than 1 week. These findings show that when implementing telemedicine and mHealth interventions for patients, the time interval should be at least 1 week in order to achieve a clinically meaningful effect on the control of systolic blood pressure.

Our research has several limitations worth discussing. First, one of the main limitations is that the duration of the interventions included in the selected studies was relatively short. There was only 1 study over 12 months, and a lack of data from studies lasting more than 12 months makes it impossible to conduct subgroup analyses. Blood pressure control in stroke survivors may be a long-term process, requiring continuous lifestyle changes. It is important to understand the long-term (over 12 months) effectiveness and safety of telemedicine and mHealth interventions in stroke survivors. Thus, more research is needed for further analyses and verification. Second, compared with usual care, the telemedicine and mHealth intervention reduced the systolic blood pressure. Statistically speaking, the difference was significant, but its clinical significance still needs to be confirmed by further study. Third, as most of the included studies only provided limited information on the profiles of the participants, it was impossible to analyze the effects of some factors on the telemedicine and mHealth interventions, such as participants’ socioeconomic and educational status and combination of drugs, which still need to be explored further in future research.

Preliminary analysis shows that the telemedicine and mHealth interventions reduced the systolic blood pressure of stroke survivors by 5.49 mm Hg on average compared with patients who received usual care. Telemedicine and mHealth interventions may be an important strategy to promote the control of systolic blood pressure in stroke survivors, and this benefit may be even greater for patients with hypertensive stroke. We also found that telemedicine and mHealth interventions with active reminders via telephone calls or SMS text messages and an intervention interval ≤1 week may be more effective. In short, telemedicine and mHealth interventions are relatively new. If used correctly, they have potential application in the control of systolic blood pressure in stroke survivors, specifically those with hypertensive stroke.

## Appendix

Multimedia Appendix 1 Search strategy.

Multimedia Appendix 2 Characteristics of the 9 randomized controlled trials included in the study.

## Abbreviations

MD: mean difference
mHealth: mobile health
PI: prediction interval
PRISMA: Preferred Reporting Items for Systematic Reviews and Meta-Analyses
RCT: randomized controlled trial
