# Supplementary material for: Effects of Telemedicine and mHealth on Systolic Blood Pressure Management in Stroke Patients: Systematic Review and Meta-Analysis of Randomized Controlled Trials
Source: JMIR Mhealth Uhealth. 2021 Jun 11;9(6):e24116. doi: 10.2196/24116 (PMC8235282; doi:10.2196/24116)
Supplement: Multimedia Appendix 1 [file mhealth_v9i6e24116_app1.pdf]

Pubmed 3082

stroke[MESH] OR brain infarction[MESH] OR transient ischemic attack[MESH] OR cerebral hemorrhage[MESH] OR subarachnoid hemorrhage[MESH]

stroke* OR cerebrovascular accident* OR CVA OR CVAs OR apoplexy OR brain vascular accident* OR brain infarction OR cerebral infarction OR transient ischemic attack* OR TIAs OR TIA OR cerebral hemorrhage* OR cerebrum hemorrhage* OR cerebral parenchymal hemorrhage* OR cerebral brain hemorrhage* OR cerebral bleeding or intracerebral hemorrhage* OR ICH OR ICHs OR intracerebral bleeding OR subarachnoid hemorrhage* OR SAH OR SAHs OR subarachnoid bleeding

Mobile Applications[MESH] OR Telemedicine[MESH] OR Text Messaging[MESH] OR Cell Phone[MESH] OR Smartphone[MESH] OR Social Media[MESH] OR internet[MESH]

mobile OR Portable Software Application OR Tele* OR mHealth OR eHealth OR e-health OR m-Health OR ?phone* OR Text* OR Short Message OR SMS OR app OR apps OR digital* OR web* OR internet* OR ?media OR wireless OR computer OR video* OR Bluetooth OR Blog* OR online OR electronic OR MP3 player OR MP4 player OR WeChat OR WhatsApp OR Twitter OR virtual reality OR interactive

voice response OR Facebook OR networking

blood pressure[MESH] OR hypertension[MESH]

blood pressure OR BP OR hypertens* OR bloodpressure OR Systolic blood pressure OR SBP OR Pulse Pressure OR PP

Cochran library 1656

stroke[MESH] OR brain infarction[MESH] OR transient ischemic attack[MESH] OR cerebral hemorrhage[MESH] OR subarachnoid hemorrhage[MESH]

stroke* OR “cerebrovascular accident*” OR CVA OR CVAs OR apoplexy OR “brain vascular accident*” OR “brain infarction” OR “cerebral infarction” OR “transient ischemic attack*” OR TIAs OR TIA OR “cerebral hemorrhage*” OR “cerebrum hemorrhage*” OR “cerebral parenchymal hemorrhage*” OR “cerebral brain hemorrhage*” OR “cerebral bleeding” or “intracerebral hemorrhage*” OR ICH OR ICHs OR “intracerebral bleeding” OR “subarachnoid hemorrhage*” OR SAH OR SAHs OR “subarachnoid bleeding”

Mobile Applications[MESH] OR Telemedicine[MESH] OR Text Messaging[MESH] OR Cell Phone[MESH] OR Smartphone[MESH] OR Social Media[MESH] OR internet[MESH]

mobile OR “Portable Software Application” OR Tele* OR mHealth OR eHealth OR e-health OR m-Health OR ?phone* OR Text* OR “Short Message” OR SMS OR app OR apps OR digital* OR web* OR internet* OR ?media OR wireless OR computer OR video* OR Bluetooth OR Blog* OR online OR electronic OR “MP3 player” OR “MP4 player” OR WeChat OR WhatsApp OR Twitter OR “virtual reality” OR “interactive voice response” OR Facebook OR networking

blood pressure[MESH] OR hypertension[MESH]
“blood pressure” OR BP OR hypertens* OR bloodpressure OR “Systolic blood pressure” OR SBP OR “Pulse Pressure” OR PP

Web of science 3338
stroke* OR “cerebrovascular accident*” OR CVA OR CVAs OR apoplexy OR “brain vascular accident*” OR “brain infarction” OR “cerebral infarction” OR “transient ischemic attack*” OR TIAs OR TIA OR “cerebral hemorrhage*” OR “cerebrum hemorrhage*” OR “cerebral parenchymal hemorrhage*” OR “cerebral brain hemorrhage*” OR “cerebral bleeding” or “intracerebral hemorrhage*” OR ICH OR ICHs OR “intracerebral bleeding” OR “subarachnoid hemorrhage*” OR SAH OR SAHs OR “subarachnoid bleeding”

mobile OR “Portable Software Application” OR Tele* OR mHealth OR eHealth OR e-health OR m-Health OR ?phone* OR Text* OR “Short Message” OR SMS OR app OR apps OR digital* OR web* OR internet* OR ?media OR wireless OR computer OR video* OR Bluetooth OR Blog* OR online OR electronic OR “MP3 player” OR “MP4 player” OR WeChat OR WhatsApp OR Twitter OR “virtual reality” OR “interactive voice response” OR Facebook OR networking

“blood pressure” OR BP OR hypertens* OR bloodpressure OR “Systolic blood pressure” OR SBP OR “Pulse Pressure” OR PP

Embase 5922

Stroke patient[MESH] OR brain ischemia[MESH] OR brain infarction[MESH] OR transient ischemic attack[MESH] OR brain hemorrhage[MESH] OR subarachnoid hemorrhage[MESH]

stroke* OR cerebrovascular accident* OR CVA OR CVAs OR apoplexy OR brain vascular accident* OR brain infarction OR cerebral infarction OR transient ischemic attack* OR TIAs OR TIA OR cerebral hemorrhage* OR cerebrum hemorrhage* OR cerebral parenchymal hemorrhage* OR cerebral brain hemorrhage* OR cerebral bleeding or intracerebral hemorrhage* OR ICH OR ICHs OR intracerebral bleeding OR subarachnoid hemorrhage* OR SAH OR SAHs OR subarachnoid

bleeding OR brain hemorrhage OR brain ischemia

Mobile Application[MESH] OR Telemedicine[MESH] OR Text Messaging[MESH] OR mobile Phone[MESH] OR Smartphone[MESH] OR Social Media[MESH] OR internet[MESH]

mobile OR Portable Software Application OR Tele* OR mHealth OR eHealth OR e-health OR m-Health OR ?phone* OR Text* OR Short Message OR SMS OR app OR apps OR digital* OR web* OR internet* OR ?media OR wireless OR computer OR video* OR Bluetooth OR Blog* OR online OR electronic OR MP3 player OR MP4 player OR WeChat OR WhatsApp OR Twitter OR virtual reality OR interactive voice response OR Facebook OR networking

blood pressure[MESH] OR hypertension[MESH]

blood pressure OR BP OR hypertens* OR bloodpressure OR Systolic blood pressure OR Pulse Pressure OR PP
