# Supplementary material for: Effects of Telemedicine and mHealth on Systolic Blood Pressure Management in Stroke Patients: Systematic Review and Meta-Analysis of Randomized Controlled Trials
Source: JMIR Mhealth Uhealth. 2021 Jun 11;9(6):e24116. doi: 10.2196/24116 (PMC8235282; doi:10.2196/24116)
Supplement: Multimedia Appendix 2 [file mhealth_v9i6e24116_app2.pdf]

Table 1. Characteristics of the 9 randomized controlled trials included in the study.

Study	Country	Study population	Mode of intervention	Control	Intervention frequency	Number		Age(Mean or median)		Sex(Female,%)		Δsystolic blood pressure(mmHg)	
						Intervention	Control	Intervention	Control	Intervention	Control	Intervention	Control
ADIE et al, 2010	United Kingdom	Hypertensive stroke or TIA[a]	Telephone	Usual care	7–10 days, 1, 2 and 4 months	29	27	-	-	-	-	0.0 19.5	-3 15
Hanley et al, 2015	United Kingdom	Stroke or TIA	Telephone	Usual care	Daily	40	15	69.9 (12.6)	73.5 (11.7)	32.5	60.0	-10.2 12.3	-3.8 13.6
Kerry et al, 2013	United Kingdom	Hypertensive stroke or TIA	Telephone support	Usual care	Daily for the first week, then weekly	168	169	71.1(12.6)	72.6 (11.4)	40.6	44.3	-1.8 21	0.9 20.5
Lakshminarayan et al, 2018	United States	Ischemic stroke or intraparenchymal hemorrhage	Smart phone	Usual care	Daily	26	24	63.1 (9.7)	68.3 (10.0)	23.1	32.0	-10.9 14.2	-5.1 16.2
MacKenzie et al, 2013	United States	Stroke or TIA	Telephone calls	Usual care	Monthly	29	27	-	-	32.1		7.2 165	-16.8 20
Sarfo et al, 2018	Ghana	Stroke	Smart phone	Standardized care	-	30	30	54.3(11.9)	55.9(13.7)	40	30	-4.0 27	-4.3 24.7
Wan et al, 2018	China	Hypertensive ischemic stroke	Telephone, short-message services	Usual care	Weekly	80	78	-	-	36.2	33.3	-9.86 15.18	-1.38 20.77
Wang et al, 2020	China	Hypertensive stroke	Telephone, short-message services	Usual care	Weekly	76	75	-	-	34.2	33.3	-10.8 12.7	-1.3 16.5
Ögren et al, 2018	Sweden	Stroke or TIA	Telephone	Usual care	4 weeks	320	340	69.9	69.3	40.6	40.9	-8.8 19.5	-3 20.8

[a]TIA: transient ischemic attack.
